# Observations on the Crural Hernia

**Published:** 1804-02-01

**Authors:** 


### Observations on the Crural Hernia, by Dr. Hull.

( Continued from our last pp. 48—59. )

7. CHOPART and Dessault, if a division of the arch be necessary, direct it to be made upwards and inwards, when the hernia is situated near the crista of the os pubis; upwards and outwards, when it is at the side of the superior and anterior spine of the os ilium, in order to avoid the epigastric artery.—*Traité des Mal. Chir.* t. 2. p. 281, 8vo. Paris, 1779.

8. Richter directs us to examine well, whether the fibres passing from the fascia lata to the crural arch be sufficiently divided before we think of cutting the arch. The direction of the incision, according to him, must depend upon the situation of the hernia: If the hernia be placed entirely, or in a great measure, on the inside of the crural vessels, so that the pulsation of the crural artery can be felt on the outside of the tumour, the incision is to be directed obliquely towards the linea alba, and is *to be made as near as possible to the inner angle of the aperture,* not only because this part is the most distant from the epigastric artery, but because the hernia especially occupies this angle: If the hernia be directly over the crural vessels, or on the outside of them, the incision should be made near the external angle of the aperture, and be directed outwards and upwards. He also advises us to make the incision of the arch, at first only sufficient to admit the introduction of a finger, by which the pulsations of the epigastric artery may be generally felt; and then, should it be necessary to enlarge the incision, this may be done in a direction where no pulsation can be felt.—*Abhandlung von den Brüchen*, ed. 2, Gottingen, 1785, and *Anfangsgründe der Wundarzneykunst*, b. 5, Gotting. 1798.

9. Else to avoid the division of the epigastric and spermatic arteries in cutting the crural arch, made a small incision through the tendon of the external oblique muscle a little above the arch; he then introduced a grooved director through this incision, and keeping its point constantly in contact with the tendon or ligament, first passed it downwards and then backwards, so as to bring it out behind and beneath the crural arch. He afterwards cut through the arch upon the director. This method was recommended by Mr. Cline, at the time I attended his lectures; and if proper care be taken, the spermatic cord will be completely out of the reach of the knife during the cutting of the crural arch, the grooved director being placed between the knife and the artery. But it is not always easy to pass the director properly beyond the posterior margin of the crural arch.

### A CASE.

Ellen Livesey, æt. 45, of Over Darwen, a village about four miles from Blackburn, had been afflicted with a crural hernia about three years, which she could always reduce by lying down on her back and pressing the tumour with her hand, before the 12th of June, 1785. On this morning she had rushed suddenly out of bed, in consequence of a knock at the door. Pain in the abdomen and sickness came on immediately. She experienced some uneasiness in the part, and attempted to reduce the hernia, but without success. She sent for a practitioner, who lived in the same village, but did not inform him of the rupture. He gave her some medicines, and these affording her no relief, Dr. Lancaster, of Blackburn, with whom I was then in partnership, was sent for the next day, the 13th. She still omitted to mention the circumstance of her having a hernia; supposing that, as she had had it so long without any material inconvenience, it could have no share in the production of her present painful and urgent symptoms. Considering the case as a colic, he directed for her a purgative mixture and clyster, and an anodyne mixture to be taken afterwards. She had no stool from the purgatives, but the pain and vomiting were relieved by taking the anodyne. Some degree of hiccup came on in the course of this day, but was not very frequent or severe. On the 14th, in the morning, the pain again increased, and I was called called in. She concealed the circumstance of the tumour till I questioned her about it. She then acknowledged it; and upon examining the tumour, I found it to be a small crural hernia. I attempted to reduce it by placing her head low and raising the thigh, at the same time pressing upon the tumour. This not answering, she was blooded, till she became faint, and another attempt was made to return the contents of the hernia, but in vain. She was next placed in the warm bath for an hour, and when she came out, a third attempt was made to replace it. This also failing, I proposed the operation. She would not consent at this time, and I returned home and directed her to take the saline mixture in the state of effervescence every three or four hours, requesting to be informed, as soon as she should consent to the performance of the operation.

On the 15th, about one in the morning, her husband came for me, and on my arrival at Darwen, having again attempted to return the hernia without success, I proceeded to operate, beginning the incision through the integuments about one inch and a half above the tumour, and carrying it down to about an inch below the tumour. The external oblique muscle and the fascia of the thigh being thus exposed, I divided the fascia, and afterwards the sac, which contained some bloody serum, and a small portion of intestine, very much altered in colour, but not mortified. I next made a small incision through the obliquus externus, rather more than half an inch above the anterior margin of Poupart's ligament; then introducing a director through this aperture I passed it downwards, with the view of getting it behind the ligament, and took care at the same time to keep the point of the director always in contact with the muscle or ligament as it passed along, for fear of its getting behind the ligamentum rotundum of the womb. The attempt to bring out the point of the director behind and beneath the ligament gave so much pain, that I desisted, and it occurred to me that I might derive some advantage from dividing the obliquus externus upon the director from the point where I had introduced it, down to the anterior margin of Poupart's ligament. I did so, and the gentleman who assisted me in the operation, was hereby enabled to raise the ligamentum rotundum out of the way of the knife, whilst I cautiously divided Poupart's ligament obliquely upwards and inwards, after insinuating the probe-pointed knife betwixt it and the sac. The division of the ligament being completed, a larger portion of intestine testine was pushed out of the abdomen, and formed a striking contrast, in point of colour, with the portion that had been strangulated. The whole of the protruded intestine was afterwards easily replaced. There was very little blood lost; and the wound being dressed, the patient was placed in bed with the breech raised. Her pain was relieved by the operation, and her pulse, which was at 126 before the operation, had now fallen to 112. I gave her an opiate and left her.

At nine o'clock the same morning I saw her again. She had slept a little, and had passed one small loose stool, so small indeed, that I could not conclude from thence that there was a free passage from the intestinal canal above the strangulated part. She had experienced no return of the vomiting. The saline mixture was continued. In the afternoon of the same day I saw her again. Her pain was now very considerable. The abdomen tumefied and tender, but not tense. An inverted peristaltic motion of the alimentary canal (differing from ordinary vomiting in this respect, that the diaphragm and abdominal muscles seemed not to be concerned in the evacuation) harrassed her almost incessantly. Every thing she took was instantly rejected. Her pulse being rather hard, though small and frequent, about twenty-four ounces of blood were taken from the arm: A strong decoction of poppy heads and chamomile flowers, with the addition of about one eighth part of common spirit, was used as a fomentation to the belly, and the boiled herbs were afterwards applied, mixed with hog's lard so as to form a cataplasm, and kept on the belly all night. A grain and a half of opium was administered every six hours in the form of a pill. The pain soon abated, and the proper action of the alimentary canal was restored.

On the 16th in the morning, I found she had slept tolerably. The tumefaction and pain of the belly were diminished; her pulse was less frequent. She had had no more stools. Eighteen ounces of blood were taken away. The opiate pill, fomentation, and cataplasm were continued.

On the 17th in the morning, she was much better in every respect. As she had not passed a stool, a purgative mixture was directed, which operated very well. She had stools regularly for some days and afterwards had a smart diarrhœa, which gave way to the combined use of opium, ipecacuanha, and catechu.

She had no bad symptoms after this time, and began to wear wear a truss in the August following, which secured her from a return of the complaint.

10. Mr. B. Bell thinks it impossible to make a free division of the crural arch in the male, without cutting the spermatic vessels across, and informs us that, instead of dividing Poupart's ligament from below upwards, he makes a slight incision into the ligament about an inch in length, beginning above and proceeding to the under edge of it; that by repeated touches with the scalpel he penetrates almost through the whole thickness of the ligament, till at last only a thin layer of it remains. And then adds, "In this situation the protruded parts may for the most part be returned with ease, as the ligament, where thus weakened by the incision, will yield gradually to the pressure applied for the reduction of the intestines."—*System of Surgery*, vol. v. p. 363, ed. 6.

From Mr. Bell's own account of the manner in which he performs the operation for the crural hernia, it appears that he only partially divides the anterior margin of the crural arch, and that he does not even touch the posterior margin. Nevertheless, Gimbernat makes the following observation upon Mr. Bell's method: "He would naturally rest the back of the scalpel upon his finger, which served as a guide to the instrument, and at the same time as a defence to the intestine. The incision being continued for an inch, the operator would inevitably cut the internal edge of the crural arch." Page 27.

11. Dr. Monro observes, "that the division of the tendon in the crural hernia is not attended with that degree of danger which some of the latest and most eminent writers have supposed, provided the edge of the knife be turned towards the umbilicus, in which direction both the epigastric artery and spermatic cord are at the greatest distance from it, and that the knife be used like a saw, dividing cautiously with it one tendinous fasciculus after another." *Description of all the Bursæ Mucosæ, &c.* p. 52, folio, Edinb.

Dr. Monro, jun. has given a more circumstantial account of the mode of operating in crural hernia, recommended by his father; from which it appears that his father first introduces a small furrowed probe, or directory, under the crural arch, before he divides it with the knife." *Observations on Crural Hernia*, p. 92, &c. Edinb. 1803.

12. Don Antonio de Gimbernat published his *Nuevo Método de Operar en la Hernia Crural*, at Madrid, in the year 1793; and Dr. Beddoes gave a translation of this work in the year 1795, under the title of *A new Method of Operating*

*ting for the Femoral Hernia*, &c. M. Alibert will not allow that the method is new. He says, " La mèthode, que M. Gimbernat propose pour le traitement de la hernie crurale, n'est pas nouvelle pour les chirurgiens Français, malgré le titre de son opuscule." *Recueil Pèriodique*, &c. tom. ix. p. 331.]

However this may be, we are indebted to Gimbernat for a more circumstantial description of the crural arch, than we before possessed, and, I believe, for the earliest publication of the method of operating, which we are now to consider. He directs us to cut that portion of the posterior margin of the crural arch, which is attached to the crista of the os pubis. His method of making the incision we shall give in the words of the Translator.

" For this purpose introduce along the internal side of the intestine a canulated or grooved sound, with a blunt end, and a channel of sufficient depth. This is to be directed obliquely inwards till it enter the crural ring, which will be known by the increased resistance, as also when its point rests upon the branch of the os pubis. Then suspend the introduction, and keeping the sound (with your left hand, if you are operating on the right side, and v. v.) firmly resting upon the branch of the os pubis, so that its back shall be turned towards the intestine and its canal to the symphysis pubis; introduce gently with your other hand into the groove of the sound a bistoury with a narrow blade and blunt end, till it enter the ring: its entry will be known as before by a little increase of resistance; cautiously press the bistoury to the end of the canal, and employing your two hands, at once carry both instruments close along the branch to the body of the pubis, drawing them out at the same time. By this easy operation you will divide the internal edge of the crural arch at its extremity, and within four or five lines of its duplicature, the remainder continuing firmly attached by the inferior band or pillar, of which it is the continuation. This simple incision being thus made without the smallest danger, the internal border of the arch, which forms the strangulation, will be considerably relaxed, and the parts will be reduced with the greatest ease." P. 45 and 46.

By this method of cutting the arch, which is well adapted to some cases of crural hernia, the stricture will be taken off without any danger of wounding the epigastric or spermatic arteries. Yet it is far from being perfectly safe. Gimbernat allows, that the urinary bladder will be liable to be wounded, if it be full at the time of the operation, and that in pregnancy of four months and upwards, the uterus uterus may also be wounded. Dr. Monro, jun. says, " If the bladder be emptied, the smaller intestines will slip downwards into the place the distended bladder of urine pre-occupied, and hence may be injured." *Observations*, p. 92.

From what has been said above respecting the course of the arteria obturatoria, when it arises by a long common trunk with the epigastrica, this artery becomes liable to be wounded. And, when there is a stricture formed by the neck of the hernial sac, or an adhesion of the sac to its contents, there would be considerable difficulty in passing the director, and great danger of wounding the intestine. On this, and indeed other grounds, it would be an improvement on Gimbernat's mode to pass the director betwixt the sac and the extremity of the crural arch, instead of introducing it within the sac.

This author says (page 12), " If the Fallopian ligament be cut across the posterior pillar of the inguinal ring it is destroyed without remedy in both sexes, and in the male an important artery," (the spermatic) " will always be cut." This, he says, has been proved by experiments made in the Hotel Dieu at Paris. But it is clear that in the methods of cutting the ligament across, suggested by Mr. Else, and practised by me on Ellen Livesey, the spermatic artery will not be injured. Again, in p. 16, he says, " if the Fallopian ligament be cut obliquely inwards, the epigastric artery is as liable to be divided as when the ligament is cut obliquely outwards, though a little further from its origin." But whoever will examine carefully the course of the epigastric artery, must be convinced that the incision of the ligament, in the former case, will be parallel to the artery, and consequently cannot affect it. Günz assures us that he performed the operation frequently on dead bodies, whose vessels were injected, and always found the epigastric artery divided, unless the incision were made obliquely towards the linea alba.

It may not be improper to observe here, that Gimbernat by restricting the signification of the term *Fallopian* ligament, and considering it as the anterior margin of the crural arch, (not as the whole arch or doubled tendon of the M. obliquus externus) may puzzle or mislead his readers. He tells us (in page 47) that the Fallopian ligament is not at all concerned in the operation he recommends, and (in page 16) he says, " Moreover, in all these methods, the Fallopian" (ligament) " is cut, which is perfectly useless, unless the incision be carried on to the internal edge of the crural arch." Now, if we consider the Fallopian ligament ligament as synonymous with the crural arch, as is frequently if not generally done, Gimbernat himself divides this ligament; and, taking it in the limited sense, which he usually does, we have very respectable authority for maintaining, that the division of the Fallopian ligament has been sufficient in many instances for removing the stricture.

13. Mr. Latta, after describing the previous steps of the operation for the crural hernia, adds, " Thus the ligament will be plainly seen, and by carefully dissecting away the fat and fascia of the thigh on each side, till you come to the insertion of the ligament, and *then dissecting it gently away from its insertion into the crest of the pubes,* the stricture will be completely removed; and this may be done without the smallest danger of cutting either the spermatic vessels in man, or the epigastric artery in woman, where these vessels are in their natural position." *System of Surgery,* vol. I. p. 281. Edinb. 1794.

Mr. Latta here directs the crural arch to be divided in the same part, in which we have found Gimbernat recommending it to be cut, and his first volume was published before Dr. Beddoes's translation. But in the two cases related by Mr. Latta, it appears, that he did not practice this method; for in the case of Mrs. H. V. he introduced a spatula under the edge of the ligament; and in the case of M. D. a grooved director, and then scratched the ligament almost completely through its whole breadth.

14. Lassus directs the incision of the crural arch to be made differently in different cases. When the hernia is situated between the femoral artery and the angle of the os pubis, the arch is to be divided upwards, and a little obliquely towards the linea alba; but, if the hernia be situated upon the femoral artery, or on the outside of it, the incision of the arch is to be made obliquely towards the os ilium. " In every case," he says, " this incision should be made as small as possible, not more than from three to four lines."—*De la Médecine Opératoire,* tome I. p. 200, &c. 8vo. *Paris,* an. 3.

15. Sabatier, when it is necessary to divide the crural arch, recommends that the incision be made in the direction of the umbilicus. He adds, " That the stricture is thus more completely taken off, because the incision falls perpendicularly on the parts which form the strangulation, and that the epigastric artery is more easily avoided."—*De la Médecine Opératoire,* tom. I. p. 150, 8vo. *Paris* 1796.

16. Ar-

16. Arneman is of opinion that the strangulation may sometimes be removed by cutting the small fibres or filaments attached to Poupart's ligament. If the crural arch must be divided, he says, the incision should be made obliquely towards the linea alba.—*System der Chirurgie*, th. I. p. 675, *Göttingen*, 1798.

17. Callisen, when other means fail of success, directs a very small incision of the Fallopian ligament to be made, and says, that extension then always succeeds. The direction of the incision is to be determined by the situation of the crural vessels. If these run along the outside of the hernia, the incision is to be made obliquely inwards; if under the hernia, or at the inner side of it, the incision is to be directed upwards and outwards.—*Syst. Chirurgiæ Hodiernæ*, t. 2. § 749. Ed. nov. 8vo. *Hafniae*, 1800.

18. Mr. Hey is satisfied that the stricture in crural hernia is not caused by Poupart's ligament, but by another, which he calls the *femoral* ligament, situated about three eighths of an inch below Poupart's. This ligament, he says, is somewhat similar to that of Poupart, but smaller, is deeper, and runs transversely, or rather ascends, as it approaches the symphysis of the ossa pubis, passing behind and decussating the extremity of Poupart's ligament.

From a careful perusal of the description and an attentive examination of the two plates which accompany it, it appears clear to me, that Mr. Hey does not understand by *femoral* ligament, the acute posterior margin of the crural arch, but a ligament situated lower on the thigh than the anterior margin of the arch. Indeed, we find him pointing out in the explanation of plate 4, letter *k*, "the femoral ligament *formed in the fascia of the thigh*, or anterior layer of the aponeurotic sheath of the great femoral vessels." He informs us, that the division of the femoral ligament may be executed without danger to the spermatic or epigastric artery, and gives the following directions for taking off the stricture. "If the tip of the finger can be introduced within the femoral ring, to guide the bubonocele knife, a small incision (for the ring is narrow) will be sufficient to set the parts at liberty. If the tip of the finger cannot be introduced at the proper place, a director with a deep groove must be used instead of the finger, but I prefer the latter. The finger, or director, should not be introduced very near the great vessels, but on that side of the intestine or omentum, which is nearest to the symphysis of the ossa pubis. *The incision may then be made directly upwards.*" p. 153. Mr. Hey therefore neither cuts the same ligament, ligament, nor in the same direction, in which Gimbernat does. Yet Dr. Monro, jun. says, "As an additional argument in favour of Mr. Gimbernat's method of operating, I may add, that it is not essentially different from that recommended and practiced by Mr. Hey of Leeds." *Observations, &c.* p. 95.

The Doctor appears to have fallen into another error, in the explanation of his own plate 3. fig. 1. He points out the portion of the posterior edge of the crural arch, comprehended betwixt the letters *e* and *d*, as the part divided by Gimbernat. But this portion of the arch appears to my eye free and unattached to the os pubis, whereas Gimbernat divides the internal foot of the arch, which is nearer the symphysis pubis even than the letter *d*.

The femoral ligament, described by Mr. Hey, and considered as distinct from the crural arch, is in my opinion a mere accidental occurrence, nothing more than a portion of the fascia of the thigh, that happens to press more strongly upon the subjacent parts than the rest. The author makes the following observation concerning this ligament in page 154: "When this is examined by dissection, it will be found to resemble the inferior border of the aponeurosis of the external oblique muscle of the abdomen. In those subjects, which I have dissected on purpose, *I have not found it equally distinct*, but it has been in all of them sufficiently apparent." I do not recollect to have met with any such ligament, either in operating for the crural hernia, in examining the bodies of two women who died of incarcerated crural hernia, or in the numerous cases, in which I have examined the crural arch of persons who have died from other causes. Since I have had the satisfaction of perusing Mr. Hey's valuable volume, I have had an opportunity of examining with considerable attention the crural arch on both sides in a female, who died of a bubonocele, yet I could not find any traces of the ligament under consideration. When a finger was passed down from the abdomen through the crural rings in this case, the principal stricture arose from the thin posterior margin of the crural ring.

From Mr. Hey's description and plates it appears to me, that where a stricture is produced by what he names the femoral ligament, this would be effectually taken off by dividing the fascia over the whole of the hernial sac up to the anterior margin of Poupart's ligament. But I know that such a division of the fascia, in some cases at least, has not proved sufficient to remove the stricture, and that it it has been necessary to cut through the crural arch to take off the strangulation, where this was not produced by the neck of the hernial sac. I am therefore induced to believe, that Mr. Hey was not always wrong in presuming formerly, that he was dividing Poupart's ligament, when he divided the part forming the stricture in crural hernia.

[ To be continued. ]

